# Have It Your Way: How Polymorphic, Injected Kinases and Pseudokinases Enable *Toxoplasma* to Subvert Host Defenses

**DOI:** 10.1371/journal.ppat.1003296

**Published:** 2013-04-25

**Authors:** John C. Boothroyd

**Affiliations:** Department of Microbiology and Immunology, Stanford University School of Medicine, Stanford, California, United States of America; University of Wisconsin Medical School, United States of America

## Introduction

As with many intracellular infectious agents, the protozoan *Toxoplasma gondii* has a quiver of effectors that it uses to co-opt host cell functions. These are injected during invasion and intersect several host pathways key to the immune response, such as STAT signaling and immunity-related GTPases (IRGs or p47 GTPases). Among the effectors so far identified, several are from a paralogous family of protein kinases and pseudokinases that are injected into the host cell from the apical secretory organelles known as rhoptries. These so-called ROP kinases are polymorphic and account for much of the difference in virulence, at least in mouse infections, found with different strains of *Toxoplasma*. How these ROPs were found and the current state of knowledge on what each does is presented in this brief review.

## 1. What Is *Toxoplasma* and What Symptoms Does It Produce?

*Toxoplasma* is a genus of obligate intracellular parasites within the phylum *Apicomplexa* [1], [2]. There is only one well-recognized species in the genus, *T. gondii*, which is found worldwide and has an enormous intermediate host range comprising almost any bird or mammal, including humans. Its definitive host (i.e., the one in which the sexual cycle occurs) is any feline. Human infection occurs either through eating undercooked meat from an infected intermediate host, like lamb, or through ingesting oocysts shed by an infected cat and present in drinking water or soil-contaminated vegetables.

The symptoms associated with *Toxoplasma* infection range from none to severe, even fatal. The factors that determine the severity include the immune status of the host and, at least in experimental animals, the particular strain initiating the infection. In humans, the data for strain-specific differences in virulence are much less clear, but several studies have suggested such an effect [3]–[5].

## 2. When and How Does *Toxoplasma* Inject Proteins into the Host Cell?

Within the invasive stages of *Toxoplasma* there are many secretory organelles [1]. One such set is the apically situated rhoptries, the bulk of whose contents are found within their bulbous base, and these are generally termed ROPs. ROPs are introduced into a host cell during invasion and traffic to the host nucleus, the nascent parasitophorous vacuole membrane (PVM), and probably other places within the infected host cell yet to be determined [6]. Despite much important work on the phenomenon, the precise mechanism by which ROPs are injected is essentially unknown.

## 3. What Comprises the ROP Cargo and What Do the ROPs Do?

Most ROP proteins so far characterized show clear homology to protein kinases [7]–[9]. Some are active kinases, while others are predicted to be pseudokinases—i.e., catalytically inactive. The best-studied active kinases are ROP16 and ROP18. ROP16 is a tyrosine kinase that mimics the action of host JAKs by phosphorylating the key tyrosine needed for activation of STATs [10]–[13]. This is an extremely rapid process such that within one minute of the commencement of invasion, STAT3 and STAT6 (at least) are activated and translocated to the nucleus where they turn on many immune response genes and downregulate the expression of others.

ROP18 is a serine/threonine kinase whose targets include the interferon-gamma-inducible p47 GTPases encoded by immune-response genes (IRGs; [14], [15]). In the absence of ROP18, IRGs can multimerize on the PVM, somehow causing it and the parasites within to be destroyed. ROP18's phosphorylation of IRGs occurs within the nucleotide-binding site, causing them to lose the ability to oligomerize and, thus, to lose activity. ROP18 also has been reported to bind and inactivate ATF6β, a transcription factor with a role in the interferon-gamma response [16]. A complete inventory of the targets of ROP16 and ROP18 has yet to be generated, but it is likely an extensive list.

The complete sequence of the *Toxoplasma* genome reveals that, in addition to ROP16 and ROP18, the ROP kinase family comprises more than 30 additional genes, many of which are under diversifying selection [9]. One of these, ROP38, is represented by three tandemly duplicated genes and appears to play a role in modulating MAPK signaling in host cells [9]. The actual target for the ROP38 kinase has yet to be determined.

Within the overall family of ROP “kinases,” most are predicted to be pseudokinases. Interestingly, these have been subject to gene duplication such that clusters of 3–12 nearly identical genes are often found at a given chromosomal location. The function of most of these pseudokinases is not yet known, but one, ROP5, has recently been shown to play a key role in the strain-specific differences in virulence in mice [17], [18]. Two nonmutually exclusive mechanisms for how ROP5 functions have been described. In the first, ROP5 binds to and thereby interferes with the oligomerization of the IRGs mentioned above [19], [20]. This both interferes with their action and makes them more susceptible to phosphorylation and, thus, permanent inactivation by ROP18. The second mechanism involves ROP5's ability to directly regulate the catalytic activity of ROP18 by allosteric means and independent of the substrate being phosphorylated [21].

Many of the ROP kinase family, including ROP5 and ROP18, are found specifically associated with the PVM. This association is mediated by an arginine-rich, amphipathic, helical domain found toward the N-terminus of the mature protein [22], [23]. At least in the case of ROP18, the proper localization provided by this domain is required for biological function—i.e., ROP18's ability to phosphorylate IRGs [22]. The N-terminal region is also necessary for ROP18's ability to bind to ATF6β [16].

## 4. How Were the ROP Kinases/Pseudokinases Discovered and What Evolutionary Pressures Led to These Strain-Specific Differences?

As stated above, strains clearly differ in their virulence in mice. Given the well-described sexual cycle of *Toxoplasma*, it is possible to cross strains by giving cats a mixed infection and then isolate and analyze the resulting F1 progeny. Such analyses revealed that mouse virulence is a multigene trait [24], [25], and several of the loci responsible for strain-specific differences in virulence have been mapped [18], [26], [27]. About the same time, microarray analysis of infected host cells revealed that strains also differ dramatically in their effects on host gene expression [28]. Hence, the *Toxoplasma* loci responsible for these differences could be mapped, as well. Collectively, these genetic studies led to the identification of ROP16, ROP18, and ROP5 as key virulence genes [18], [26], [27], and ROP16 was simultaneously identified as also a key mediator of host gene expression [28].

A small number of strains have come to dominate in many regions [29], [30], raising the question of what evolutionary forces might have led to their emergence. One attractive model is that different strains evolved for different host species and that allelic differences in the various “virulence” factors are a reflection of similar differences in their respective targets in those hosts. A priori, this could be a difference between host species such that *Toxoplasma* strain A carries versions of ROP5, ROP16, and ROP18 that are perfectly “tuned” to the IRGs, ATF6β, and STATs of, say, a mouse, while strain B carries alleles of these ROPs that are optimized for the corresponding molecules in a sparrow. This possibility has been tested by comparing the different efficiencies with which ROP16s of different strains activate their STAT targets in mammals (mice and humans) vs. birds (chickens). The results showed that, contrary to the model, the strain-specific differences in ROP16's effect were independent of host species, at least in vitro [10]! This led to a friendly amendment to the model—i.e., that the differences between the hosts that the strains have evolved to infect might be in the environment experienced by that host rather than the species of host, per se. For example, it could be that strain A evolved in hosts that were simultaneously infected with worms or microflora that pushed the immune system in a particular direction, while strain B evolved in hosts that differed in this key respect. This could explain why one version of ROP16 can drive a macrophage toward the classical, inflammatory state of activation, while another pushes them toward the alternative, wound-healing state [31]; a host population that is chronically infected with worms is expected to be more in the latter state, and this might select for a strain of *Toxoplasma* that pushes against this to ensure there is a balanced response to the parasite. If such a strain infected a worm-free population of hosts, i.e., one that is tilted more toward an inflammatory (Th1-type) response, the result might be a hyperinflammatory cytokine storm that was lethal to the host, resulting in little if any transmission of the parasite.

## 5. What Is the Significance of the ROPs to Human Health and Our Understanding of Host-Pathogen Interactions in General?

Clinically, most *Toxoplasma* infections in humans result in little if any disease. If the exceptions to this scenario are due to strain type, knowing the mechanism responsible for the severe disease would enable us to treat the infection appropriately. For example, infection with a strain that drives a hyperinflammatory infection might best be treated by a regimen that includes steroids or other anti-inflammatory agents. Such treatment in a patient with a strain that already down-modulates inflammation might be disastrously counterproductive.

Most of the ROPs remain mysterious in their function. In fact, it is mostly only the ones that have a major, strain-specific impact on virulence or gene expression that have so far had functions ascribed. This has led to discovery of an intersection with previously known host pathways like those mediated by STAT or IRG proteins. It is likely that the nonpolymorphic ROPs have equally important functions and very possible that the targets of these are not pathways that are currently understood in the immunology world. Proteins like the diverse NOD-like receptors (NLRs) that are presumed to function in innate immunity but a majority of which have no known ligand are but some of many possible targets for the yet-to-be-studied ROPs. *Toxoplasma* has had millennia to evolve an impressive array of effectors, and they are likely far more “aware” of host immune defenses than are we.
